# A promising antitumor method: Targeting CSC with immune cells modified with CAR

**DOI:** 10.3389/fimmu.2022.937327

**Published:** 2022-08-11

**Authors:** Binjie Huang, Lele Miao, Jie Liu, Jiaxing Zhang, Yumin Li

**Affiliations:** ^1^ Department of General Surgery, Second Hospital of Lanzhou University, Lanzhou, China; ^2^ Key Laboratory of the Digestive System Tumors of Gansu Province, Second Hospital of Lanzhou University, Lanzhou, China

**Keywords:** CSC, immunotherapy, targeted strategy, CAR, CAR-NK

## Abstract

Tumors pose a great threat to human health; as a subgroup of tumor cells, cancer stem cells (CSCs) contribute to the genesis, development, metastasis, and recurrence of tumors because of their enhanced proliferation and multidirectional differentiation. Thus, a critical step in tumor treatment is to inhibit CSCs. Researchers have proposed many methods to inhibit or reduce CSCs, including monoclonal antibodies targeting specific surface molecules of CSCs, signal pathway inhibitors, and energy metabolic enzyme inhibitors and inducing differentiation therapy. Additionally, immunotherapy with immune cells engineered with a chimeric antigen receptor (CAR) showed favorable results. However, there are few comprehensive reviews in this area. In this review, we summarize the recent CSC targets used for CSC inhibition and the different immune effector cells (T cells, natural killer (NK) cells, and macrophages) which are engineered with CAR used for CSC therapy. Finally, we list the main challenges and options in targeting CSC with CAR-based immunotherapy. The design targeting two tumor antigens (one CSC antigen and one mature common tumor antigen) should be more reasonable and practical; meanwhile, we highlight the potential of CAR-NK in tumor treatment.

## 1 Introduction

According to global cancer statistics, there were approximately 19.3 million new cancer cases and 10.0 million cancer deaths in 2020. The global cancer burden continues to increase and is expected to be 28.4 million (a 47% rise from 2020) in 2040 (1). Therefore, cancer remains a serious threat to human health and safety. An increasing amount of antitumor strategies has been proposed to reduce the damage caused by tumors. Nonetheless, the present results require optimization. During cancer research, scientists found a particular tumor cell cluster, cancer stem cells (CSCs), which harbor the properties of stem cells and common tumor cells simultaneously; these include self-renewal and multidirectional differentiation (2, 3). Subsequently, CSCs contributed to the genesis, development, metastasis, and recurrence of tumors because of their special properties (4). In addition, CSCs are resistant to conventional chemotherapy and radiotherapy *via* many different mechanisms, such as abnormal reactive oxygen species (ROS) scavenger levels, increased adenosine 5′-triphosphate (ATP)-binding cassette efflux transporter expression, increased autophagic activity, and decreased ferroptosis (5, 6).

In order to reveal the mechanisms with which CSCs contribute to tumor development, more detailed information of CSCs has been reported. Researchers have developed many methods to identify and isolate CSC, including multiparametric flow cytometry (FCM), magnetic activated cell sorting (MACS), side population (SP) sorting, sphere formation assay, chemotherapeutic drugs, immunoselection, and density gradient centrifugation; meanwhile, they have suggested that the unique features of CSC including intracellular enzyme activity, the concentration of reactive oxygen species, the mitochondrial membrane potential, promoter-driven fluorescent protein expression, autofluorescence, suspension/adherent culture, cell division rate, resistance to cytotoxic compounds, or hypoxia and invasiveness/adhesion can also be used to identify CSC (7). In addition to conventional methods to identify CSC, the recent single-cell technologies have also proven promising in identifying CSCs populations, CSC biomarkers, and the range of tumor microenvironment cellular constituents that contribute to tumor growth (8).

Research has found that CSCs harbor a unique mitotic pattern (asymmetric and symmetric division) (9) and a unique metabolic phenotype (oxidative phosphorylation (OXPHS) and aerobic glycolysis) (10). Additionally, CSCs can be marked by many biological molecules in different tumor types. Inhibiting CSCs remains a crucial step in cancer treatment. Many methods have been used to inhibit or reduce CSCs until now, including monoclonal antibodies targeting particular surface molecules of CSCs (CD44, CD133, epithelial cell adhesion molecule (EpCAM), aldehyde dehydrogenase (ALDH), etc.), signal pathway inhibitors (wingless/integrated (Wnt)-β-catenin, hedgehog interactive (Hh), janus kinase and signal transducer and activator of transcription (JAK-STAT), etc.), and energy metabolic enzyme inhibitors, inducing differentiation therapy and immunotherapy (tumor vaccine, oncolysis virus, immune checkpoint inhibitor, chimeric antigen receptor (CAR) therapy, etc.). Among all the immunotherapy options, immune cells engineered with CAR show potential for CSC-targeted therapy.

In recent years, CAR-T cell therapy has been successful against many tumor types, especially hematological malignancies. Using CAR with immune effector cells could make them more accurate and effective in targeted cell elimination, which is an ideal strategy to inhibit CSCs. With the development of CAR therapy, different CAR designs and modified immune effector cells have been developed for tumor therapy. Although more options in CAR application continue to emerge, many issues remain to be addressed in CAR-based therapy for CSC elimination.

## 2 CSC targets in immunotherapy

Although there are various methods to kill tumor cells, most targets used in immunotherapy are tumor-associated antigens, not tumor-specific antigens. Therefore, the lack of effective tumor-specific targets remains a crucial problem, which also exists in CSC elimination. The most representative molecular markers are CD44 and CD133, expressed in the CSCs of many tumor types, and have been used as therapeutic targets for CSC elimination. In addition, researchers have found many other new molecular targets **(** **Table 1** **)** which could improve effectiveness and contribute to CSC therapy.

**Table 1:** CSC markers which have been used as therapeutic targets in different tumors.

Tumor types	CSC target	Reference
**Hematological malignance**		
Leukemia	CD90, CD34, CD123, CLL-1, ALDH, CD38	(11–16)
**Solid tumor**		
Liver cancer	CD44, CD133, CD90, EpCAM, ALDH, CD13, OV-6, α2δ1, ICAM-1	(17–23)
Pancreatic cancer	CD44, CD133, CD73, DCLK1, CXCR4, ABCB1, STAT3, CD47	(24–30)
Gastric cancer	CD44, CD133, CD24, CXCR4, ALDH, EpCAM, LGR5	(31–37)
Breast cancer	CD44, CD133, CD29, CD90, DLL1	(38–41)
Breast cancer	CD44, CD133, LGR5	(42–44)
Urinary cancer	CD44, CD133, CD105	(45–47)

DLL1, delta-like canonical Notch ligand; STAT, signal transducer and activator of transcription; CXCR4, C–X–C chemokine receptor type 4; LGR5, leucine-rich repeat containing G protein-coupled receptor 5; ICAM-1, intracellular adhesion molecule 1; DCLK1, doublecortin-like kinase 1; CLL-1, C-type lectin-like molecule-1; OV-6, oval cell marker antibody; ABCB1, ATP-binding cassette subfamily B member 1.

## 3 CAR therapy in CSC treatment

### 3.1 Development of the CAR

**Figure 1:**
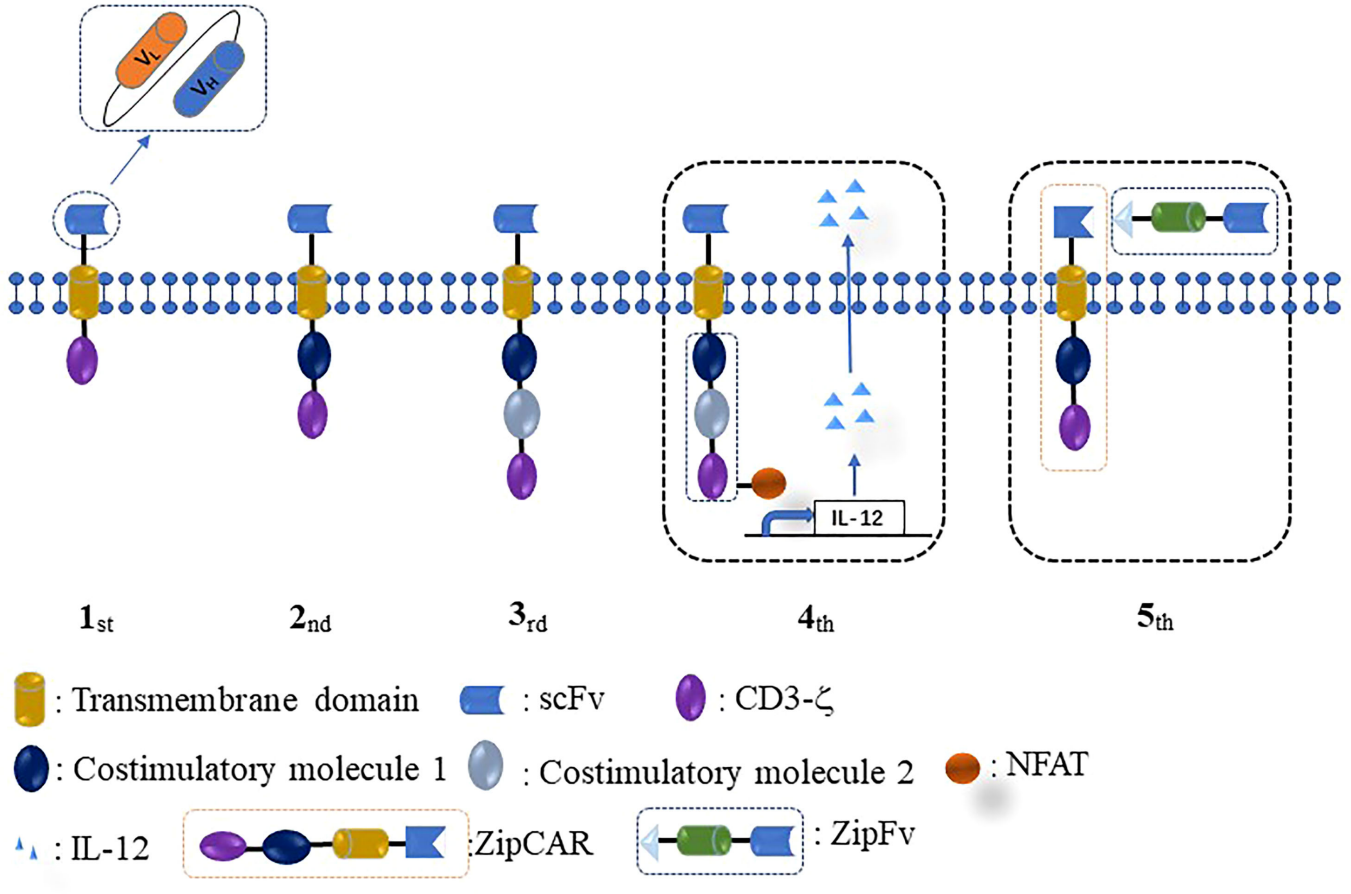
Different generations of CAR design. The intracellular domain of the first CAR only consists of one signaling domain (CD3ζ). The second CAR adds one co-stimulatory molecule (CD28/4-1BB/OX40/ICOS) to the intracellular domain of the first CAR. The third CAR contains two costimulatory molecules in the intracellular domain. The fourth CAR is designed by adding NFAT or suicide gene based on the second or third CAR. The fifth CAR uses a “third-party” intermediate system to separate the antigen-binding domain of CAR from the T-cell signaling unit.

The initial conception of CAR-T was proposed by an Israeli scientist in 1993. This first-generation CAR consisted of three parts, a single-chain fragment (ultracellular domain), a trans-membrane domain, and CD3ζ (intracellular domain) (**Figure 1**). The first CAR-T was ineffective for tumor therapy because it lacked persistent proliferative capacity in the human body. Thus, the second CAR was designed by adding a co-stimulatory molecule CD28/4-1BB into the intracellular domain of the first-generation CAR. Subsequently, to achieve stronger and more persistent proliferative activity, the third-generation CAR was designed by adding two co-stimulatory molecules (CD28/4-1BB and CD28/OX40/4-1BB) into the first CAR. The fourth-generation CAR was designed to improve the efficacy, potency of antitumor ability, and therapeutic safety by adding a nuclear factor of the activated T-cell (NFAT) or suicide gene besides targeting tumor cells. Recently, the conception of fifth-generation universal CAR (BBIR CAR, SUPRA CAR) was proposed by researchers. Fifth-generation CAR has several advantages over rigid CAR structures, such as convenience to switch the antigen target without modification of CAR structure, more controllable activities or toxicities of CAR therapy, and multiple choices in effector cell types and signaling domains (48).

### 3.2 Immune effector cells modified with CAR in CSC therapy

#### 3.2.1 CAR-T in CSC therapy

The classic case of immune cells engineered with CAR is the T cell, which is artificially modified into a CAR-T cell to target the CD19 molecule on the human leukemia cell. Since then, many CAR-T cells designed with different tumor targets and CAR generations have been tested in many tumor types, including hematological malignancies, glioblastoma, melanoma, liver cancer, gastric cancer, and pancreatic cancer (49–54). Five CAR-T products with two different targets (CD19: Kymriah, Yescarta, Teacarus, Breyanzi; BMCA: Abecma) were approved to be applied to clinical cases by the Food and Drug Administration (FDA) in 2017 and 2021. As a subgroup of tumor cells, CSCs play a more crucial role in tumor development. Therefore, the prognosis can be improved by targeting CSC with CAR-T therapy (55, 56).

##### 3.2.1.1 Anti-CD133-CAR-T

CD133 is a well-known surface marker of CSC in many different tumors, and researchers have indicated that targeting CD133 with CAR-T is a feasible way to inhibit CSCs. Xuekai Zhu et al. verified the eliminating effect of anti-CD133-CAR-T cells against AC133+ glioblastoma stem cells (GBM-SCs). This research found that anti-CD133-CAR-T cells showed excellent killing capability against AC133+ patient-derived GBM-SCs and a glioma mouse model (57). CSCs survive conventional chemotherapy because of their multidrug resistance. Yang Han observed upregulated CD133 expression in a human gastric cancer sample and the BGC-823 cell line after cisplatin treatment; these researchers designed the anti-CD133-CAR-T cell to verify its anti-CSC capability. Finally, they indicated that the combinational strategy of cisplatin and anti-CD133-CAR-T could inhibit gastric cancer progression in three different xenograft models, improving the outcome by targeting normal and CSCs simultaneously in gastric cancer (31). In another study, Thanich Sangsuwannukul et al. designed the fourth-generation anti-CD133-CAR-T cells; they found that the cholangiocarcinoma stem cells marked by CD133 could be lysed in a dose-dependent manner (58). A similar antitumor effect was also observed in hepatocellular carcinoma stem cells (NCT02541370) (59).

##### 3.2.1.2 Anti-EpCAM-CAR-T

EpCAM, also named CD326, ESA, or EGP40, is a transmembrane glycoprotein encoded by GA-733-2, whose molecular weight is approximately 40 kDa. EpCAM plays a crucial role in cell-to-cell and cell-to-cellular matrix adhesion. EpCAM is a commonly used surface marker to identify CSCs in many tumor types, including gastric cancer, pancreatic cancer, liver cancer, and other cancers. Several EpCAM-related CAR-T strategies have been proposed to provide promising approaches for eliminating CSCs. Juan Fu et al. designed the third-generation anti-EpCAM-CAR-T cell, composed of EpCAM-scFv, a CD8 transmembrane domain, a CD8/4-1BB costimulatory domain, and an intracellular CD3ζ; they found that anti-EpCAM-CAR-T showed prominent killing capability against EpCAM highly expressed ovarian cancer tissue and a cell line (SKOV3). The antitumor ability of this method was significantly higher than the phosphate-buffered saline (PBS) group and NC-T group in SKOV3-CDX models (60). Yan Zhou et al. indicated that EpCAM was overexpressed in five colon cell lines and designed anti-EpCAM CAR-T cells as a promising strategy to inhibit CRC development (61). In addition, anti-EpCAM-CAR-T-related preclinical evaluations have been conducted and shown potential in tumor therapy (62).

#### 3.2.2 CAR-NK in CSC therapy

The natural killer (NK) cell plays a vital role in the human body’s antitumor immunity regulation, virus infection control, and immunoregulation. Unlike T and B cells, NK cells show non-specific cytotoxicity without being sensitized. NK cells could also be reprogrammed with CAR into CAR-NK to inhibit tumor development.

According to previous studies, CAR-NK cells have also been applied in CSC-targeted strategies. Rüdiger Klapdor et al. designed the third-generation anti-CD133-CAR-NK92 cells and found that anti-CD133-CAR-NK92 cells could inhibit ovarian tumor cell development; the most important result was that the sequential treatment strategy consisting of cisplatin followed by CD133-CAR-NK92 cells showed a stronger killing effect than cisplatin or CD133-CAR-NK92 cells alone (63). These researchers designed another dual CAR-NK cell targeting CD24 and mesothelin at the same time. This strategy was effective against ovarian cancer cells by targeting CSCs and common tumor cells simultaneously (64). In colorectal cancer, Qing Zhang et al. designed the second-generation EpCAM-CAR-NK92 cells and indicated that EpCAM-CAR-NK92 harbored a high potential in inhibiting CRC development. The combinational strategy consisting of EpCAM-CAR-NK92 and regorafenib could enhance the anti-CRC effect in mouse models (65).

#### 3.2.3 CAR-macrophage in CSC therapy

A core problem in immunotherapy of solid tumors is the infiltration rate of effector cells in the tumor microenvironment. Researchers have found that macrophages are the main innate immune cells and harbor the highest infiltration rate in the tumor microenvironment, which makes them a potential target for tumor therapy. Many macrophage-based studies have proven their success in inhibiting tumor development (66). Researchers have found that macrophages can also be engineered with CAR to target tumor antigens and improve solid tumor therapy. Michael Klichinsky et al. found an adenoviral vector to overcome the genetic editing resistance of primary human macrophages and endow the edited macrophages with sustained pro-inflammatory phenotypes (M1). These CAR macrophages, overexpressed pro-inflammatory cytokines, and chemokines had enhanced antigen presentation processes and resistance to immunosuppressive cytokines. Moreover, these cells showed potential against humanized solid tumor mouse models (67). Li Zhang edited induced pluripotent stem cell (iPSC)-derived macrophages with CAR and found that the expression of CAR on macrophages enhanced phagocytosis of tumor cells and anticancer activity (68). Macrophage therapy is a potential avenue in immune cell-editing-based immunotherapy, especially against solid tumors. However, we did not find any CAR-macrophage-related studies with CSC therapy during our literature search. Therefore, this could be a promising direction in future research.

### 3.3 Current challenges and options for targeting CSC with CAR-cells

#### 3.3.1 Challenges for targeting CSC with CAR cells

##### 3.3.1.1 Off-target effect

An ideal target is necessary for immunotherapy, and it should be abundantly and specifically expressed on tumor cells. A non-specific target will cause serious side effects because of non-specific immunization. CAR therapy overcomes several obstacles in tumor immunotherapy; the most groundbreaking one is the major histocompatibility complex (MHC) unrestricted recognition of tumor antigens. However, many issues remain, including off-target activity. Most targeted antigens in immunotherapy are tumor-associated antigens (TAA). Finding a specific antigen to target specific tumor cells, such as the prostate-specific antigen for prostatic cancer, can be challenging. Additionally, the antigens used for CAR therapy are surface antigens on tumor cells, which further limits the choices for CAR therapy. Nonetheless, certain CSC makers are surface antigens, which could be targeted by CAR therapy.

##### 3.3.1.2 Antitumor activity of immune effector cells edited by CAR

Several methods, including additional costimulatory domains in different CAR, have been used to enhance the expansion and persistence of effector cells in patients. Nonetheless, low antitumor activity remains a core problem for several reasons, including the source of immune cells.

The optimal immune effector cell for CAR therapy should be autogenous to enhance immune tolerance and persistence in the acceptor. However, many disadvantages exist in autogenous CAR therapy, such as T-cell dysfunction due to exhaustion and senescence, high manufacturing costs, and a delay in treatment for patients. Thus, allogenic immune cells from healthy hosts have been commonly used in CAR-T therapy. As exogenous antigens, CAR-T cells will cause immune reactions and be rapidly eliminated by the patient’s immune system, and this causes graft versus host disease (GVHD) and low antitumor activity due to the short persistence of CAR-T cells in the patient system (69). Compared to T cells, NK cells modified with CAR exhibit advantages in many aspects, such as tumor cell CAR-independent killing ability (NCRs, NKG2D, CD226, and ADCC), reduced alloreactivity, and the existence of mature cell lines (NK92) (70).

##### 3.3.1.3 CRS and ICANS

Cytokine release syndrome (CRS) refers to the strong inflammatory response state caused by many pathogenic factors in humans, which mainly occurs in immunotherapy. CRS is the main side effect of CAR-T. Many kinds of cytokines and chemokines, such as interleukin (IL)-6, IL-8, IL-10, and monocyte chemoattractant protein-1 (MCP-1), will be released by activated CAR-T cells and other immune cells, leading to high fever, hypotension, and even life-threatening multiorgan dysfunction. Researchers have tried to reduce CRS *via* various methods. The most effective way to reduce CRS is with cytokine and chemokine inhibitors. For instance, many IL-6 inhibitors (tocilizumab, sarilumab) have been used to reduce CRS, including CAR-T/coronavirus disease of 2019 (COVID-19)-related CRS (71, 72). Another way to reduce CRS is through a novel CAR molecule design. Zhitao Ying et al. generated a new anti-CD19 CAR molecule (CD19-BBz (73)) by using a tertiary-structure-prediction program (Phrye2) and altering sequences encoding the extracellular and intracellular domains of the CD8α molecule. These researchers found that the new anti-CD19-BBz (73) CAR-T was safer because of its lower levels of cytokines, higher levels of antiapoptotic molecules, and slower proliferation rate (74). Immune effector cell-associated neurotoxicity syndrome (ICANS) is the second most common side effect of CAR-T therapy. ICANS is characterized by several mental symptoms, such as aphasia, word-finding difficulty, seizures, and coma. Several variables were independent predictors of ICANS severity during CAR-T treatment, including bone marrow disease, cyclophosphamide, fludarabine lymphodepletion, and CAR-T cell dose and peak expansion (75). To achieve safer CAR-T therapy, researchers have established different grading and corresponding handling principles for CRS and ICANS (76). Additionally, research suggests that patients can benefit from the earlier and potentially more targeted interventions during treatment.

Moreover, there should be less CRS and ICANS with CAR-NK therapy. However, this hypothesis requires extensive research through preclinical and clinical trials, especially for solid tumors (77).

#### 3.3.2 Options for targeting CSC with CAR-cells

##### 3.3.2.1 Suitable CAR choice

Optimizing the CAR structure is a viable way to improve the CAR therapy’s accuracy, efficiency, and safety. Although different generations of CAR overcome several obstacles with their special design, many new problems were found in CAR therapy, especially in solid tumors. Thus, researchers continue to design new specialized CAR structures to utilize the limited antigens on targeted cells. Meijia Yang et al. designed a tandem CAR which could target two tumor antigens (CD70, CD276); these tandem CAR-T cells exhibited enhanced cytolysis and cytokine release in the tumor cells expressing CD70 and CD276 (78). Mohammadmahdi Sabahi et al. proposed a tandem AND-gate CAR-T cell which utilized a combination of a modular synthetic Notch receptor (synNotch) and a tandem CAR-T cells to target glioblastoma CSCs (79). Additionally, some other special CARS, such as dual-signaling CARs, inhibitory CARs, AND-NOT CARs, CARs with three scFvs, ON/OFF-switch CARs, and universal CARs, have been proposed and proven their success and advantages in tumor therapy (80). However, these CARs mainly target common tumor cells, not CSC. Future research should apply CARs with different structures to CSC therapy.

##### 3.3.2.2 Suitable effector cell choice

Although many issues remain with CAR-T therapy, T cells are the most important immune effector cells in humans and the most mature and used cell types in CAR-based immunotherapy. According to current data, CAR-NK cells show many advantages over CAR-T cells. Firstly, CAR-NK cells show more tolerance and less CRS. There is more than one NK-cell origin for CAR-NK production, such as periphery (PB), umbilical cord blood (UCB), human embryonic stem cells (HESCs), induced pluripotent stem cells (iPSCs), and mature NK cell lines. On the one hand, autogenous NK cells could avoid GVHD and reduce the cost of immunotherapy. The NK cell lasts for less than 20 days in the human body, and the short persistence can reduce the level of immune response and avoid serious CRS. Secondly, in addition to their specific killing ability against tumor cells, CAR-NK cells can also exhibit antitumor capability because of their natural nonspecific killing ability through the cytotoxic receptor. Thirdly, the sources of NK cells are diverse, of which the mature NK92 cell line harbors several advantages. NK92 cells are easy to amplify, are easy to transfect, and elicit low rejection levels.

The main immune cell harboring CAR to target CSC is the T cell; CAR-NK is rarely used for CSC therapy **(** **Table 2** **)**. Recent reports have shown that CAR-NK cells appear more viable, effective, and safer than CAR-T cells. However, CAR-NK cells require further validation through preclinical and clinical trials before they can be applied for CSC therapy in tumor patients.

**Table 2:** Recent studies about CAR-T/NK in CSC therapy.

Year	Author	CSC target	Immune cell	Tumor	References
2015	Zhu et al.	CD133	T cell	Glioblastoma	(57)
2015	Deng et al.	EpCAM	T cell	Prostate cancer	(81)
2017	Vita Golubovskaya	CD47	T cell	Pancreatic cancer	(82)
2018	Song et al.	CD44/Her-2	T cell	Gastric cancer	(83)
2018	Wang et al.	CLL-1	T cell	AML	(14)
2018	An et al.	CD38	T cell	Myeloma	(84)
2019	Hu et al.	CD133	T cell	Glioma	(85)
2019	Zhou et al.	EpCAM	T cell	Solid tumor	(61)
2019	Zhang et al.	EpCAM	T cell	CRC	(62)
2021	Anna Stornaiuolo et al.	CD44	T cell	Solid tumor	(73)
2021	Fu et al.	EpCAM	T cell	Ovarian cancer	(60)
2021	Nian et al	EpCAM	T cell	AML	(86)
2017	Rüdiger Klapdor et al.	CD133	NK92 cell	Ovarian cancer	(63)
2019	Rüdiger Klapdor et al.	CD24	NK92 cell	Ovarian cancer	(64)
2019	Zhang et al.	EpCAM	NK92 cell	CRC	(87)

AML, acute myelocytic leukemia; CRC, colorectal cancer.

##### 3.3.2.3 Suitable targeted antigens choice

**Figure 2:**
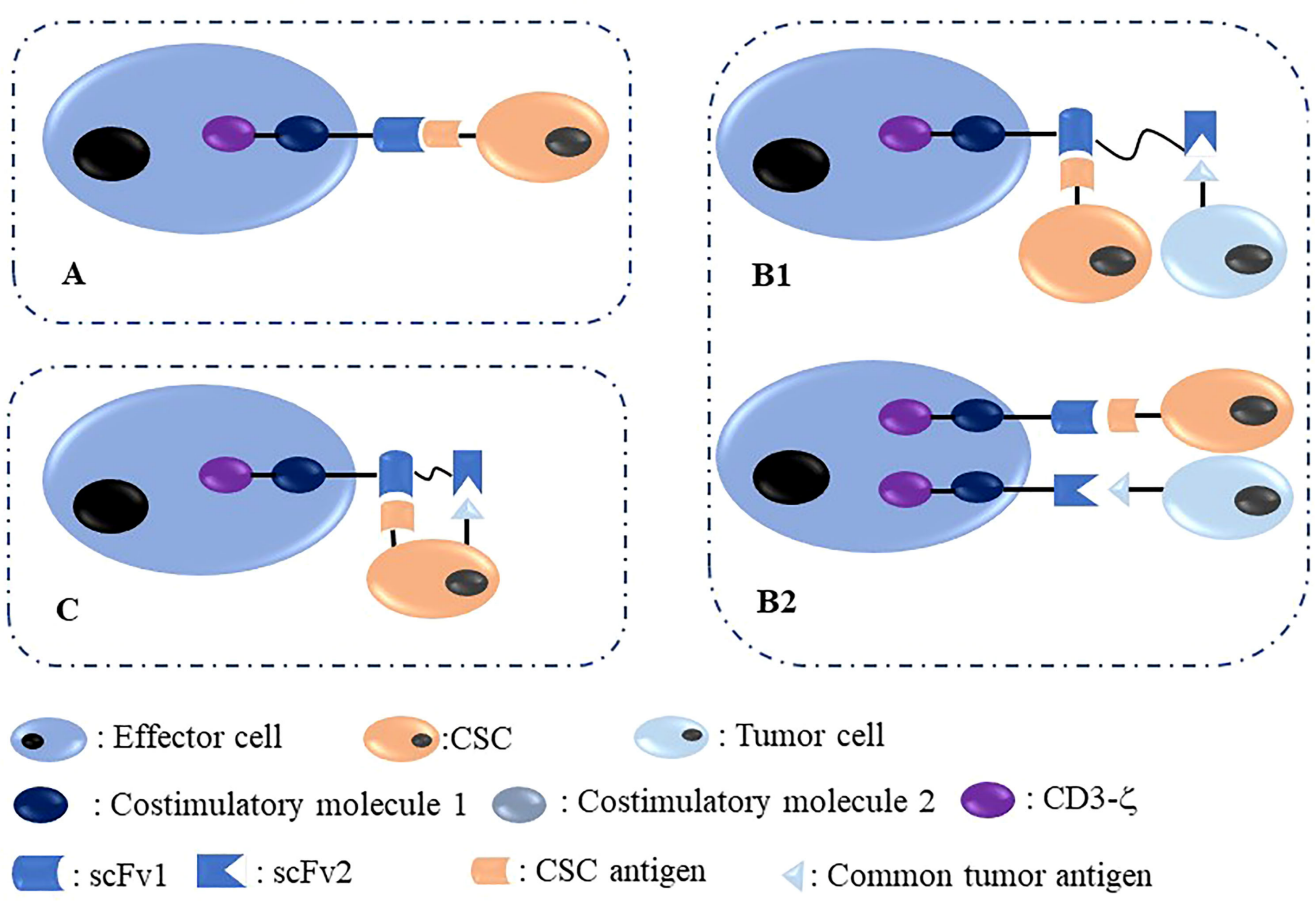
Different methods to target CSC with immune effector cells modified with CAR. **(A)** One target CAR-effector cell targeting a CSC antigen located on CSCs; **(B)** Dual-target CAR-effector cell targeting two different antigens, including one common tumor antigen and one CSC antigen located on common tumor cells and CSCs, respectively. **(C)** Dual-target CAR-effector cell targeting two different antigens, including one common tumor antigen and one CSC antigen located on one CSC simultaneously.

CSCs are marked with several biological molecules, including surface molecules such as CD44, CD133, CD24, and EpCAM, which could be utilized as targets to inhibit CSCs through several targeting methods, including CAR therapy. The core problem is that CSCs are difficult to target because of their small percentage and heterogeneity characterized by unstable surface antigen expression. Nonetheless, the development of CAR-based immunotherapy has an enhanced capability to target CSCs. In **Figure 2**, we summarized targeted antigen choices for CAR-T/NK in CSC elimination; of these, the methods shown in panels **B1** and **B2** are more reasonable and effective.

## 4 The current status of clinical trials of CAR-based immunotherapy in CSC therapy

To evaluate the clinical therapeutic effect of CAR-based immunotherapy in CSC inhibition, researchers have designed several CAR-T cells targeting some mature CSC markers to validate their efficacy in phase I/II clinical trials. As shown in **Table 3**, most of the current status of these clinical trials is recruiting, and part of them have been terminated for different reasons. The only completed clinical trial was conducted at the Biotherapeutic Department and Pediatrics Department of Chinese PLA General Hospital, which started on 1 June 2015 and ended on 1 September 2017. Researchers designed CD133 CAR-T cells to treat 21 patients with advanced hepatocellular carcinoma (HCC). They observed the promising antitumor activity and a manageable safety profile in CD133 CAR-T therapy, the median OS was 12 months (95% CI, 9.3–15.3 months), and the median PFS was 6.8 months (95% CI, 4.3–8.4 months). During treatment, one patient had a partial response, 14 had a stable disease for 2 to 16.3 months, and six progressed after T-cell infusion; they found that hyperbilirubinemia was the most common high-grade adverse event and several circulating molecules, such as endothelial growth factor (VEGF), soluble VEGF receptor 2 (sVEGFR2), stromal cell-derived factor (SDF)-1, and interferon (IFN)-γ, could be the potential biomarkers of CD133 CAR-T therapy in HCC (59). In addition, we did not find any CAR-NK-related clinical trials in CSC therapy. The conclusion of whether clinical patients can benefit from these CAR-based immunotherapies remains to depend on more completed clinical trial results.

**Table 3:** The current clinical trials with CAR therapy targeting CSCs.

Target	NCT	Phase	Current status	Condition	Year
CD44	NCT04427449	I/II	Recruiting	Cancers (CD44v6^+^)	2020
	NCT04097301	I/II	Terminated	AML, MM	2019
CD133	NCT03473457	NA	Terminated	AML	2018
	NCT02541370	I/II	Completed	AM	2015
CD123	NCT04014881	I	Recruiting	AML	2019
	NCT04599543	I	Not yet recruiting	AML	2020
	NCT02937103	I/II	Unknown	MM	2016
	NCT04265963	I/II	Recruiting	AML	2020
	NCT03190278	I	Recruiting	AML	2022
EpCAM	NCT02915445	I	Recruiting	Nasopharyngeal/Breast cancer	2016
	NCT03563326	I	Recruiting	Gastric cancer	2018
	NCT05028933	I	Recruiting	Digestive system cancers	2022
	NCT02729493	NA	Unknown	Liver cancer	2016
CD117	NCT03473457	NA	Terminated	AML	2018
	NCT03356782	I/II	Recruiting	Sarcoma	2017
ROR1	NCT05274451	I	Recruiting	Solid tumors	2022
	NCT02706392	I	Terminated	Cancers (ROR1^+^)	2016
CXCR4	NCT04727008	I	Not yet recruiting	MM	2021

AM, advanced malignancies; MM, multiple myeloma; NA, not available; ROR1, receptor tyrosine kinase-like orphan receptor 1.

## 5 Discussion

Inhibition of CSCs plays a crucial role in antitumor therapy. Several different methods to target CSCs have been proposed in previous studies, including monoclonal antibodies, signal pathway inhibitors, energy metabolism inhibitors, differentiation inducers, and immunotherapy. CAR-based immunotherapy has proven its success in hematologic malignancies and several solid tumors. With the development of CAR, more effective and safer CAR designs have been used in antitumor therapy; meanwhile, it allows modified immune effector cells (T cells, NK cells, etc.) to target more than one tumor surface antigen at the same time, further improving efficacy in reducing CSCs and common tumor cells. In addition to T cells, the NK92 cell line has been used in CAR therapy. Therefore, the choices of immune effector cell could be more diversified in the future.

Furthermore, several issues deserve attention in CSC-CAR therapy. First, CSCs are also tumor cells and harbor small percentages and unstable surface antigen expression. As shown in **Figure 2**, CAR therapy consists of a mature common tumor antigen and a CSC surface antigen is more effective in CAR-based antitumor therapy, making the CSC-targeted strategy a “second sword”. Second, CSC surface markers need to be detected before CSC-targeted therapy. Third, more novel and effective CSC-related surface antigen molecules should be developed in the future. Fourth, a combinational strategy consisting of CAR therapy and conventional chemotherapy could be more effective in antitumor treatment.

Obviously, inhibition of CSCs is of great significance in tumor treatment; CAR-based therapy provides a new potential way to target CSCs. Particularly, CAR-NK cells exhibit more advantages than CAR-T cells. Therefore, these CAR-based immunotherapy options may comprise novel anti-CSC research and therapy in the future.

## Author contributions

BH and LM: Original draft preparation, investigation, and figure preparation. JZ and JL: Investigation, language assistance. YL: Methodology, supervision. All authors approved the submitted version.

## Funding

This work was funded by the Special Research Project of Lanzhou University Serving the Economic Social Development of Gansu Province (054000282), Lanzhou Talent Innovation and Entrepreneurship Project (2020-RC-38), Major Science and Technology Special Project of Gansu Province (20ZD7FA003), and The Applied Research of c-Met and PD1/CD28 Fusion Receptor CAR-T in Gastric Cancer (751000–054000002).

## Acknowledgments

The authors thank professor YL for his comments on this manuscript.

## Conflict of interest

The authors declare that the research was conducted in the absence of any commercial or financial relationships that could be construed as a potential conflict of interest.

## Publisher’s note

All claims expressed in this article are solely those of the authors and do not necessarily represent those of their affiliated organizations, or those of the publisher, the editors and the reviewers. Any product that may be evaluated in this article, or claim that may be made by its manufacturer, is not guaranteed or endorsed by the publisher.
